# Financing Maternal Health and Family Planning: Are We on the Right Track? Evidence from the Reproductive Health Subaccounts in Mexico, 2003–2012

**DOI:** 10.1371/journal.pone.0147923

**Published:** 2016-01-26

**Authors:** Leticia Avila-Burgos, Lucero Cahuana-Hurtado, Julio Montañez-Hernandez, Edson Servan-Mori, Belkis Aracena-Genao, Aurora del Río-Zolezzi

**Affiliations:** 1 Center for Health Systems Research, National Institute of Public Health, Morelos, Mexico; 2 Research Center for Evaluation and Surveys, National Institute of Public Health, Morelos, Mexico; 3 National Center for Gender Equity and Reproductive Health, Ministry of Health, Mexico City, Mexico; Georgia State University, UNITED STATES

## Abstract

**Objective:**

To analyze whether the changes observed in the level and distribution of resources for maternal health and family planning (MHFP) programs from 2003 to 2012 were consistent with the financial goals of the **related** policies.

**Materials and Methods:**

A longitudinal descriptive analysis of the Mexican Reproductive Health Subaccounts 2003–2012 was performed by financing scheme and health function. Financing schemes included social security, government schemes, household out-of-pocket (OOP) payments, and private insurance plans. Functions were preventive care, including family planning, antenatal and puerperium health services, normal and cesarean deliveries, and treatment of complications. Changes in the financial imbalance indicators covered by MHFP policy were tracked: (a) public and OOP expenditures as percentages of total MHFP spending; (b) public expenditure per woman of reproductive age (WoRA, 15–49 years) by financing scheme; (c) public expenditure on treating complications as a percentage of preventive care; and (d) public expenditure on WoRA at state level. Statistical analyses of trends and distributions were performed.

**Results:**

Public expenditure on government schemes grew by approximately 300%, and the financial imbalance between populations covered by social security and government schemes decreased. The financial burden on households declined, particularly among households without social security. Expenditure on preventive care grew by 16%, narrowing the financing gap between treatment of complications and preventive care. Finally, public expenditure per WoRA for government schemes nearly doubled at the state level, although considerable disparities persist.

**Conclusions:**

Changes in the level and distribution of MHFP funding from 2003 to 2012 were consistent with the relevant policy goals. However, improving efficiency requires further analysis to ascertain the impact of investments on health outcomes. This, in turn, will require better financial data systems as a precondition for improving the monitoring and accountability functions in Mexico.

## Introduction

In recent decades, countries around the world have identified improving maternal health as a policy objective given its critical relevance in the reduction of social inequalities [1,2]. Mexico is no exception: at the beginning of the 21st century, its maternal mortality ratio reached 74.1 deaths per 100,000 live births, with an unacceptable inverse correlation between the distribution of this indicator and economic development across all 32 states [3,4]. Like those in many other countries, Mexico’s policy makers therefore began to formulate more effective MHFP policies.

In line with its commitment to achieving the Millennium Development Goals (MDGs), the Mexican government undertook to cut its 1990 maternal mortality ratio by three-quarters by 2015 [2,4]. In 2001, it launched the *Arranque Parejo en la Vida* (Fair Start in Life) program. One of the major challenges encountered by this program in improving access to skilled childbirth care and strengthening family planning in those rural areas with the highest maternal mortality rates [4,5] was the need to coordinate the efforts of the numerous health institutions that make up the fragmented, employment-based and decentralized Mexican healthcare system.

Prior to the healthcare system reform in 2003, only formal-sector workers and government employees could access social security services. Because of supply and budget restraints, those without social security had limited access to government services and were forced to purchase healthcare from private providers [6–8]. Out-of-pocket (OOP) payments accounted for half of total health expenditure, and nearly 3% of Mexican households reported catastrophic health spending [6,9]. Before the reform, the population with social security received twice as much money as the population without, and distribution of per capita spending among State Health Services (known as *SESAs* from the initials in Spanish) varied as much as fivefold. [10,11]

**Fig 1:**
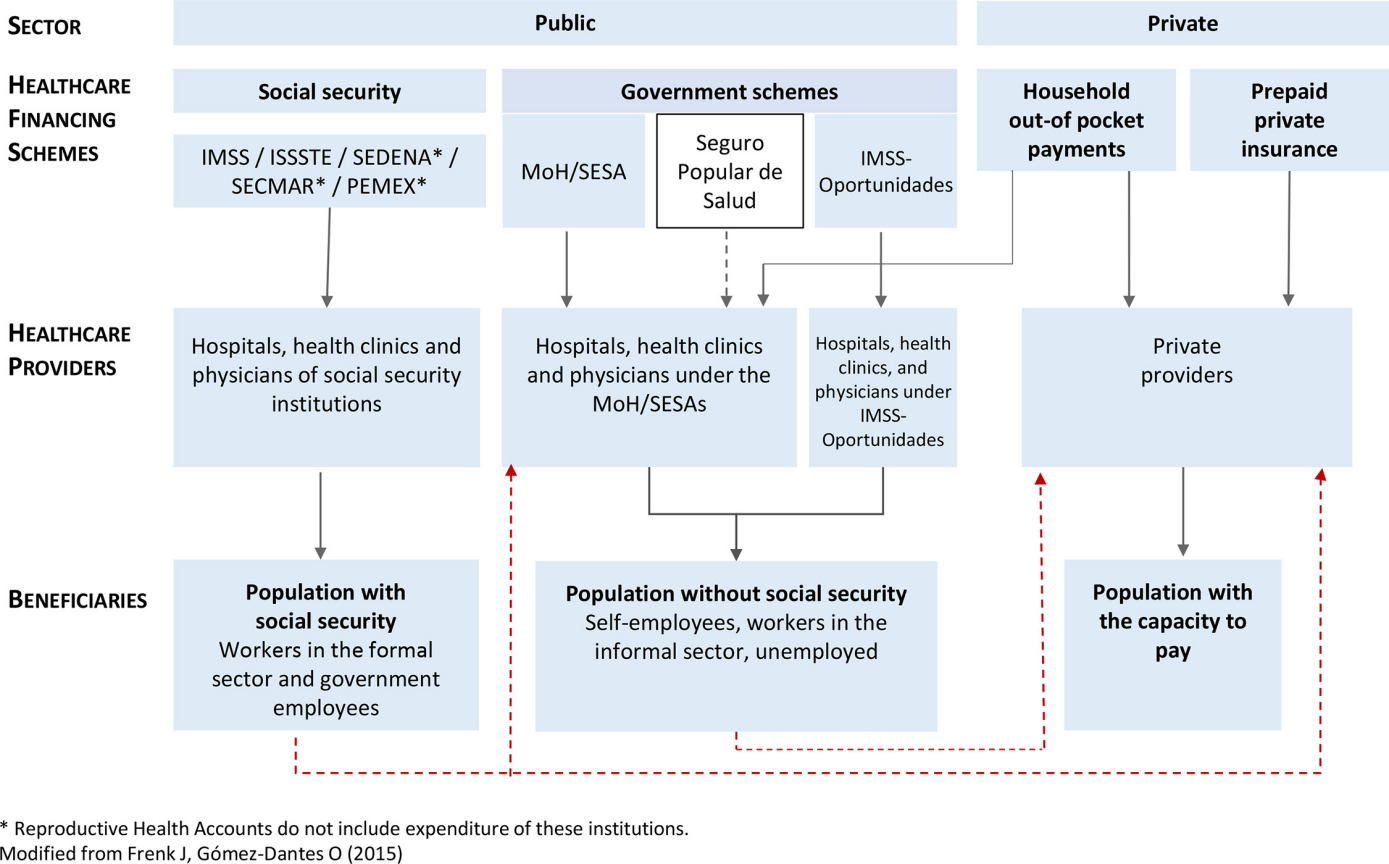
The Mexican Health System. The Mexican health system is fragmented and labor-based. It includes a public and a private sector. The public sector consists of two sub-sectors: (a) social security, which comprises the Mexican Institute for Social Security (IMSS); the Institute for Social Security and Services for Civil Servants (ISSSTE); and social security institutions for the army, the marines and the national oil company workers (SEDENA, SECMAR and PEMEX). Social security coverage went from 38.3% in 2000 to 38.9% in 2012; and (b) government schemes (restricted by user fee), which include the Ministry of Health, the State Health Services, the Seguro Popular (since 2004); and the IMSS-Oportunidades program. Until 2003, access to providers was limited, leaving beneficiary population without the capacity to pay out of this public health sub-system. The Seguro Popular, designed to remove this barrier, has opened the access to health services for 38.5% of the Mexican population. (c) the private sector, which comprises household out-of-pocket payments and prepaid private insurance. Private providers offer services to those with the capacity to pay, including the population with and without social security.

To reduce these gaps and ensure financial protection for the poorest households, the government launched the System of Social Protection in Health (SSPH) [8,12], as a mechanism for reducing OOP spending by increasing public expenditure [8,12,13]. Its core component, the *Seguro Popular* (*SP*), is a voluntary public healthcare insurance scheme, primarily for those lacking social security (Fig 1). The *SP* introduced changes in the allocation of new resources to *SESAs* on the basis of capitation payments [8,11,12], thus tying expenditure to potential demand (affiliated population), and weakening the traditional dependence on budget allocation.

MHFP policies were aligned with these wider healthcare reforms in pursuit of the MDGs. Mechanisms were established to remove financial barriers and offer women increased access to healthcare, particularly for pregnancy, childbirth and family planning [4,5]. One such mechanism was the *Embarazo Saludable* (Healthy Pregnancy) strategy, introduced in 2008, which allowed pregnant women without social security to enroll in *Seguro Popular* [5]. The Comprehensive Strategy for Reducing Maternal Mortality [5], launched in 2009, catalyzed the participation of public health institutions in the delivery of emergency obstetric care to anyone in need.

Monitoring all these changes was fundamental to informing policy development and tracking financial progress towards policy commitments. Distribution of health expenditure data solely by general categories, however, did not allow for financial analysis of specific health areas such as maternal health and family planning. A set of Reproductive Health Subaccounts (RHS) was therefore constructed in line with the Health Accounts System, and has been in place since 2004 [14,15]. After a full decade of continual estimates, these subaccounts offer a detailed description of national and state-level expenditure on reproductive health by financial scheme, function (activity/program) and beneficiary, thus providing national and state-level perspectives on reproductive health funding. The aim of this study was to analyze whether the changes observed in the level and distribution of resources for MHFP were consistent with the financial objectives of the policies implemented in Mexico from 2003 to 2012.

### Reproductive Health Subaccounts framework

The RHS in Mexico were designed in line with the Organization for Economic Co-operation and Development’s System of Health Accounts framework [16] and the World Health Organization’s Guide to Producing Reproductive Health Subaccounts [17]. They are thus methodologically compatible with the System of National Accounts adopted by the United Nations [18].

Consumption is the main axis for financial tracking. For MHFP, consumption refers to all transactions concerning the provision and final utilization of MHFP healthcare goods and services within health systems [19]. Its analysis considers healthcare functions, namely antenatal, childbirth (vaginal or cesarean) and postpartum services; treatment of complications during pregnancy and childbirth, such as miscarriages; and family planning health activities [16].

The second analytical dimension is *health providers*. These are the organizations and actors dealing in healthcare goods and services, such as hospitals offering inpatient and outpatient care, as well as ambulatory healthcare suppliers. Under the RHS, countries allocate money to their health systems through an array of financial arrangements known as *health financing schemes* [16,17]. These constitute the third analytical dimension.

Fig 1 illustrates the basic healthcare financing arrangements in Mexico: (a) social security, including the Mexican Social Security Institute (*IMSS*, from its initials in Spanish), and the Institute of Social Security and Services for Civil Servants (*ISSSTE*); (b) government schemes, including spending by the Ministry of Health (MoH), *SESAs*, *IMSS*-*Prospera* and SSPH; (c) OOP payments; and (d) private insurance. These financing arrangements target specific populations or beneficiaries, representing the fourth analytical dimension of the RHS. MHFP beneficiaries include all women of reproductive age (WoRA), those aged 15–49 years [17,20].

## Materials and Methods

### Sources of information

Data from the RHS 2003–2012 series were analyzed using RHS matrices designed to break down actual spending by public/private and national/sub-national schemes dealing with reproductive health functions.

Public spending estimates considered RHS national and state-level data on Mexican health accounts, budgetary information, and services provided by health-related public institutions (e.g., the MoH, *SESAs*, social security organizations and the SSPH) [21]. To identify the volume of MHFP health services provided between 2003 and 2012, information was analyzed for a total of 44.4 million hospital discharges, 176.2 million hospital inpatient days, and 774.9 million general and specialty visits.

Private spending estimates drew upon RHS data available only at the national level for private insurance expenditure reported by the Mexican Association of Insurance Institutions [22], and OOP expenditure for health purposes published biannually by the National Survey on Household Incomes and Expenses [23]. In alternate years where surveys were not conducted, OOP spending was estimated on the basis of data from the previous year, adjusted for inflation [24]. For OOP expenditure, the RHS distinguish between households with and without social security protection by examining whether or not WoRA or the heads of households reported affiliation to a social security scheme. Further details on the methodology for calculating public and private spending can be found in Avila et al [15, 25].

The number of beneficiaries by financing scheme was determined by applying the coverage percentages calculated by the MoH [26] to official population projections [27].

### Measurement and data analysis

A longitudinal descriptive analysis was performed on the levels and trends in MHFP expenditure in Mexico from 2003 to 2012. Expenditure was first deflated using the accumulated inflation figures published by the National Institute of Statistics (*INEGI* in Spanish) [24]. It was then converted into 2012 international dollars using a purchasing power parity rate of 7.99 Mexican pesos per international dollar [28].

To track whether changes in MHFP expenditure were aligned with maternal health policy objectives, the following financial indicators were employed [5,6,16,17,29]:

1. Public and OOP spending, as percentages of total MHFP expenditure. Increased public expenditure coupled with reduced OOP spending over time was taken to reflect a reduction of financial barriers in accessing MHFP services.
2. Average public expenditure per WoRA. By using this indicator to compare health financing schemes, it was possible to assess whether the financial imbalance between populations with and without social security had decreased.
3. Public expenditure on complications as a percentage of expenditure on preventive care. This indicator relates to the goal of providing women with greater access to health services for pregnancy, childbirth and family planning. Reductions in this indicator reflect greater increases in public expenditure on preventive care than on the treatment of complications. Preventive care included antenatal and postpartum care, as well as family planning services provided by ambulatory providers.
4. OOP expenditure on specific MHFP health functions: (a) childbirth (vaginal or cesarean), (b) complications of pregnancy or childbirth, (c) antenatal care, and (d) family planning. The disaggregation of these indicators by households with and without social security allowed determination of whether OOP spending had fallen more in households without social security.
5. Measurement of the alignment of MHFP expenditure with potential service demand. Spearman correlations were calculated between the logarithms of public expenditure at state level and the number of WoRA by health financing scheme for the years 2003 and 2012.
6. Public MHFP expenditure per WoRA was calculated at the state level. Median values were compared using K-sample tests, and reductions in financial imbalances were examined over time by calculating maximum/minimum ratios.

All analyses were performed using Stata 13.1 software [30]. This project was approved by the Research, Ethics and Biosecurity Committees of the National Institute of Public Health.

## Results

**Fig 2:**
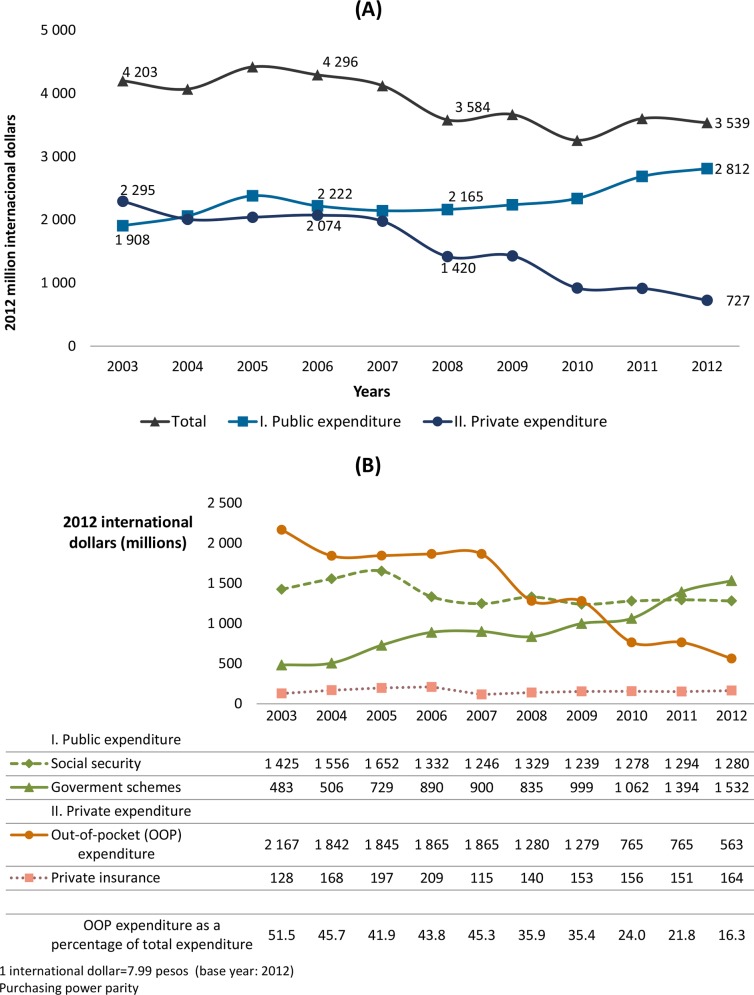
MHFP expenditure by financing scheme, 2003–2012. (A) Total, public and private MHFP expenditure. (B) MHFP expenditure by financing scheme. Public expenditure rose over the period analyzed because of a dramatic growth in government scheme spending. By contrast, private expenditure fell as a corollary of the drop in OOP spending.

Public expenditure on MHFP varied over the 10-year period analyzed, ending in 2012 with a total increase of 47% versus 2003. By contrast, private spending decreased from 2005; by 2012, it had fallen to approximately one-third of the 2003 level. As a percentage of total MHFP expenditure, public spending increased from 45.4% in 2003 to 79.0% in 2012 (Fig 2A).

From 2003 to 2012, public expenditure rose because of a dramatic increase in spending on government schemes. By contrast, during the same period, private expenditure declined as a corollary of lower OOP spending.

Overall government expenditure rose markedly (by roughly 317%) for this period. Allocations increased for the social security population until 2005, and then fell by approximately 20% over the following year, remaining fairly constant for the remainder of the period analyzed. The sharpest drop occurred in OOP expenditure, which fell from 50.2% of total MHFP expenditure in 2003 to just under 16% in 2012, a drop of 74% (Fig 2B).

**Fig 3:**
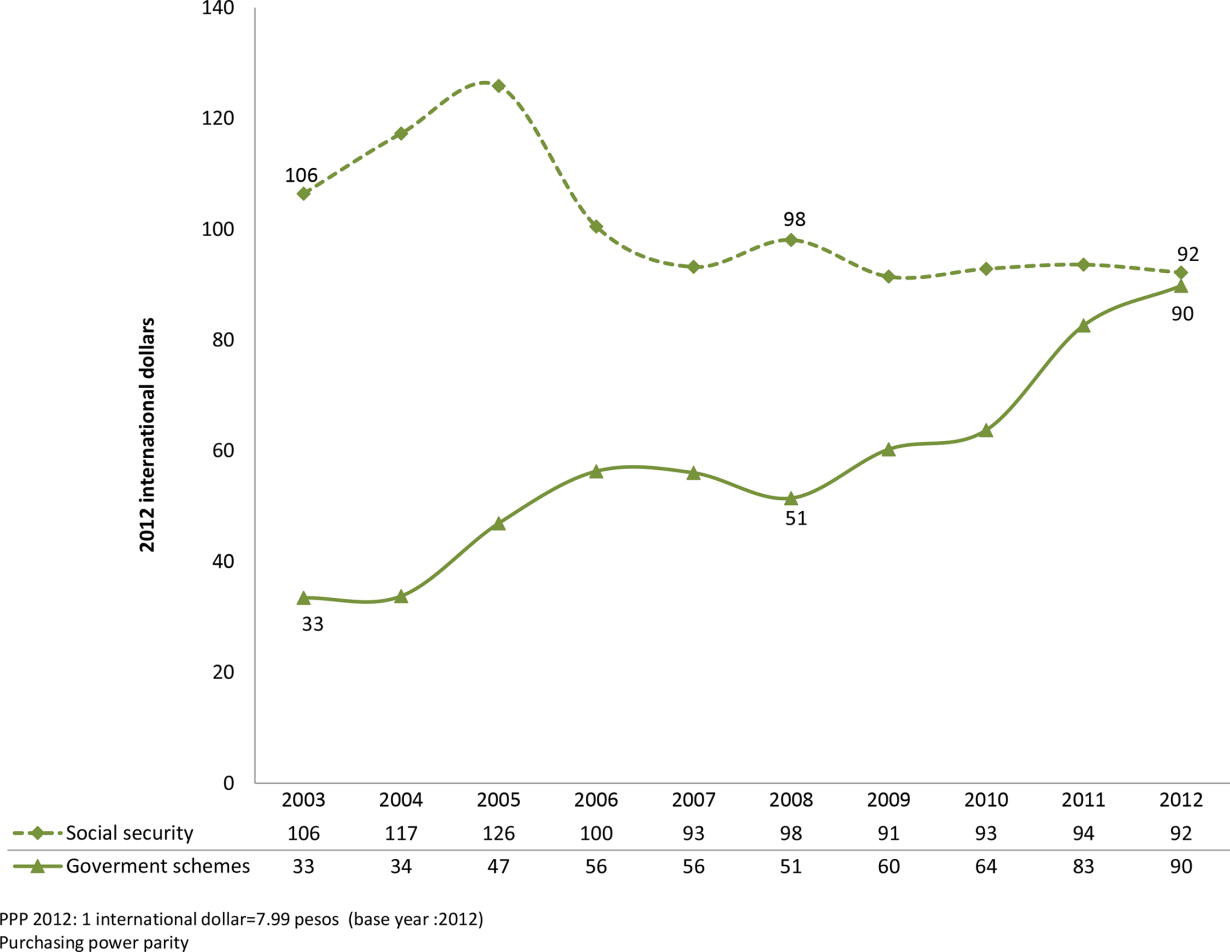
Public expenditure on MHFP by financing scheme per WoRA, 2003–2012.

Between 2003 and 2012, national expenditure on social security per WoRA decreased by 13%, but increased almost threefold on the other government schemes. As a result, the ratio of the difference in expenditure between social security and other government schemes dropped from 3.18 in 2003 to only 1.03 in 2012 (Fig 3).

**Fig 4:**
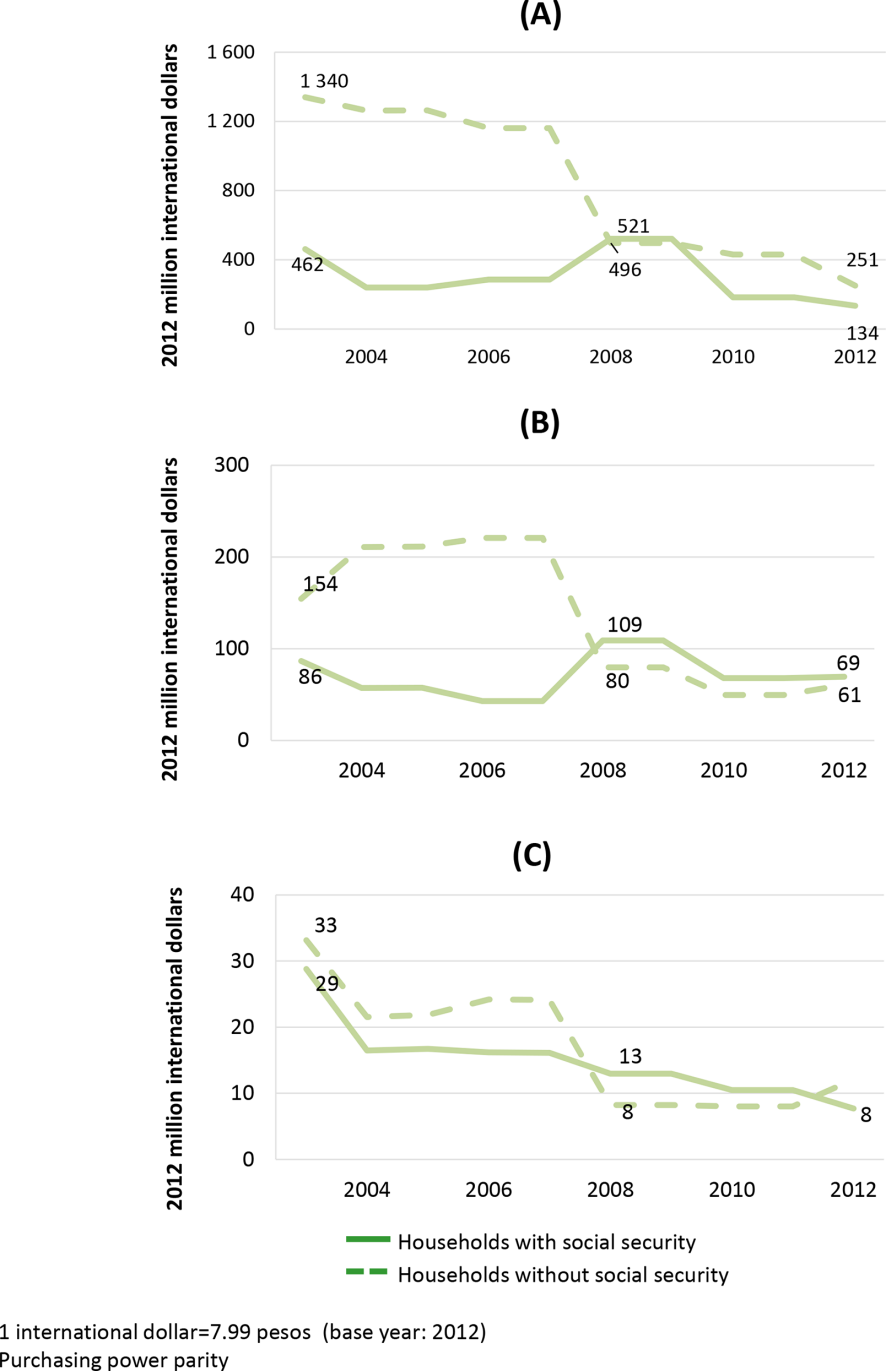
Public MHFP expenditure by health function, 2003–2012.

During the period analyzed, public expenditure on treatment of complications increased, with 2012 levels 37% higher than in 2003 (Fig 4). Public expenditure on preventive care rose between 2003 and 2006, dropped again to a low point in 2008–2009, concluding in 2012 with an increase of 62% over 2003 figures. As a result, public expenditure on complications dropped from 178.5% of expenditure on preventive care in 2003 to 150.7% in 2012 (Fig 4).

**Fig 5:**
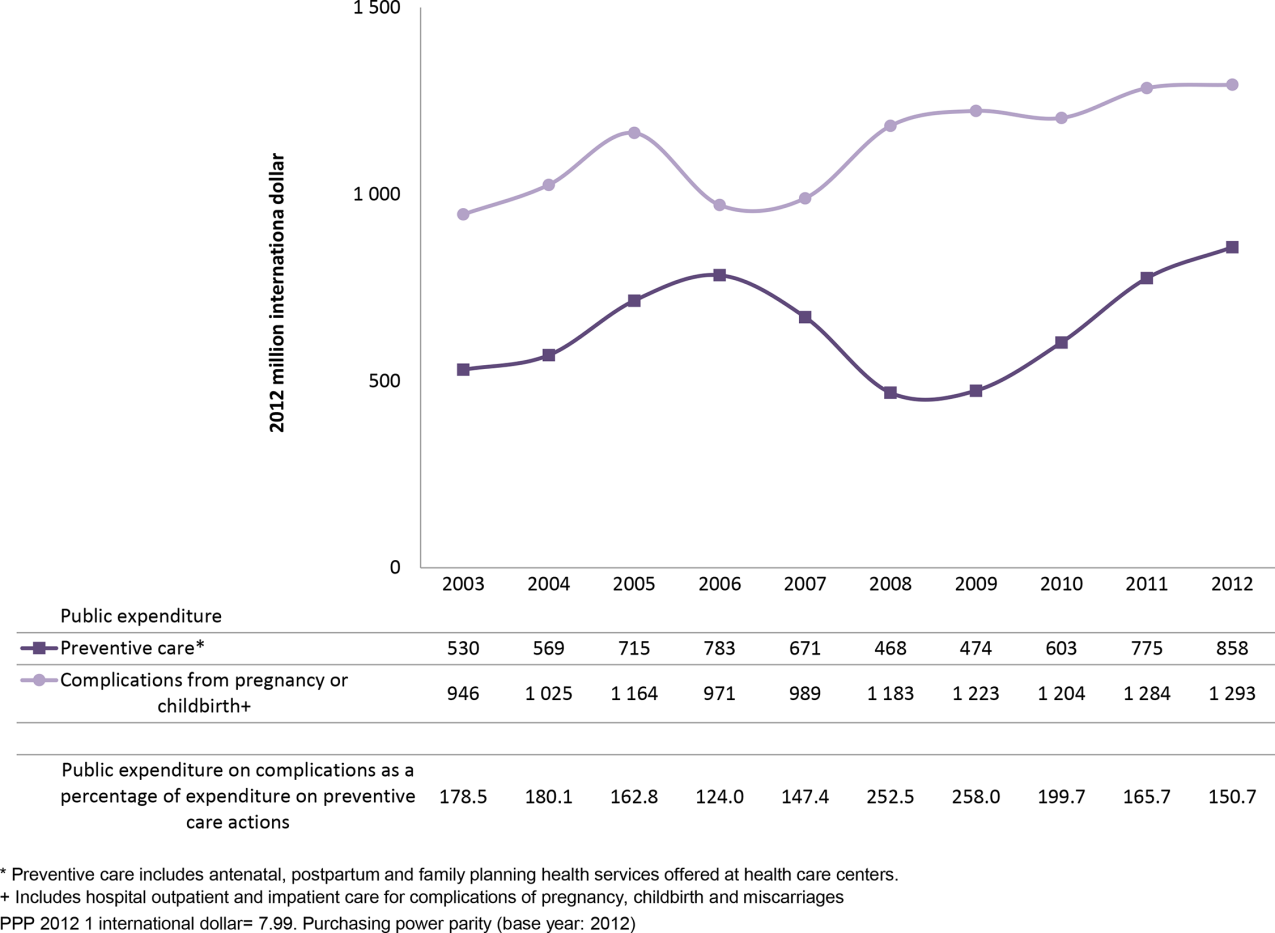
Out-of-pocket expenditure on selected health functions, 2003–2012. (A) Childbirth (vaginal or cesarean) as well as pregnancy and childbirth related complication. (B) Antenatal care. (C) Family planning. Across functions, the decrease of OOP expenditure in households without social security was greater than that in other households.

Fig 5 shows OOP expenditure on selected health functions by households with and without social security. For households without social security, 2003–2012 saw a steady decrease in OOP spending on childbirth and complications, representing an 81% reduction over 2003 expenditure. OOP expenditure in these households also decreased by 61% for antenatal care, and 64% for family planning services. Expenditure by households with social security on childbirth and complications, and antenatal care continued to fall steadily until 2007. It then began to rise, peaking in 2008, and then reducing again, concluding in 2012 with reductions of 71% and 20%, respectively, over 2003. OOP expenditure on family planning dropped progressively throughout the period analyzed, reaching a total decrease of 73% by 2012.

Across health functions, except family planning, OOP expenditure for households without social security dropped more substantially than for households with social security.

In 2003, expenditure by government schemes on MHFP at the state level showed a moderate association with the potential demand (Spearman’s rho = 0.65; p = 0.00) (Panel 6a). Throughout the 10 years analyzed, this association increased (Spearman’s rho = 0.75; p = 0.00), with average expenditure on government schemes rising significantly at the state level (χ^2^ = 9.9; p = 0.00).

**Fig 6:**
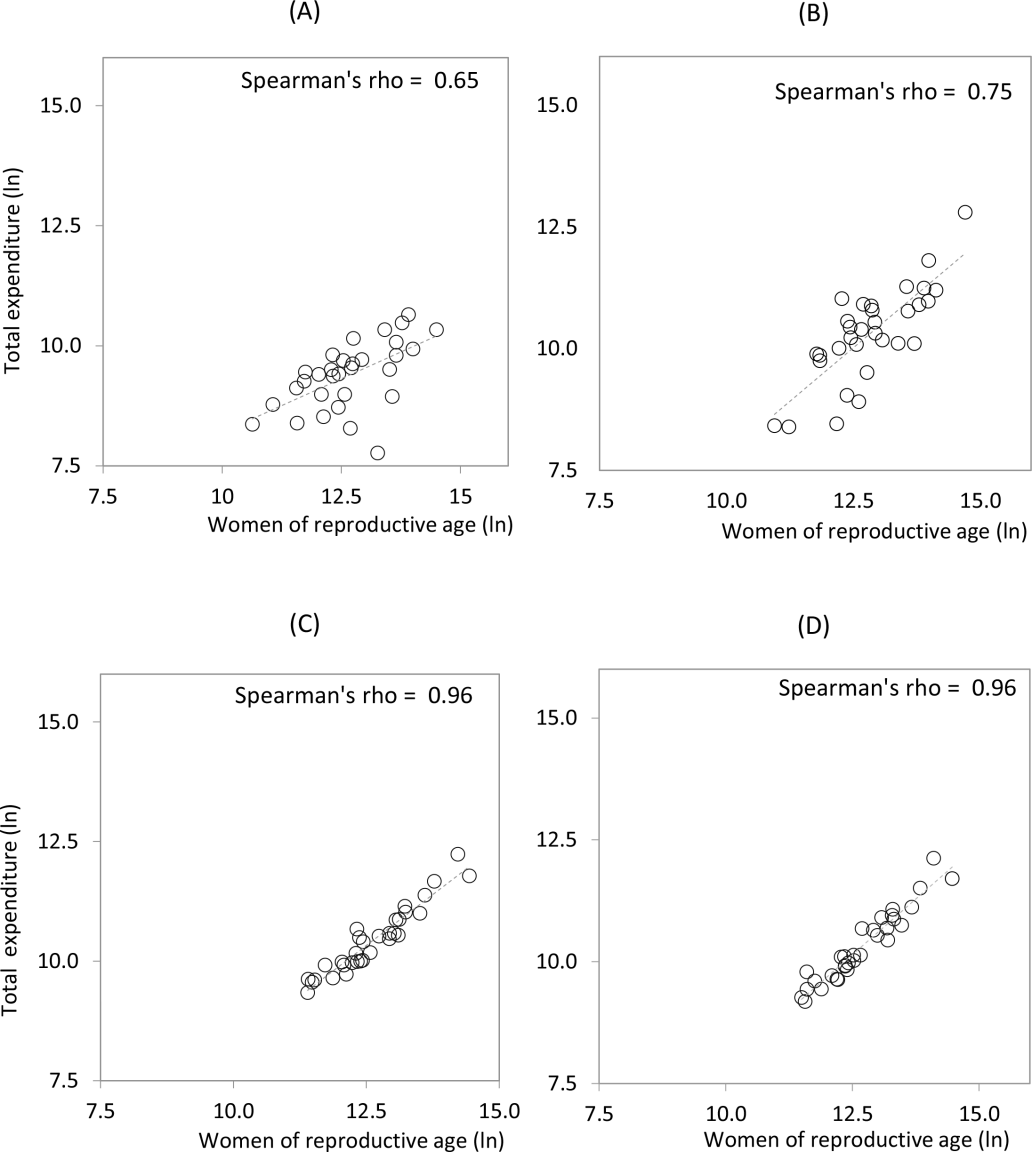
Public expenditure by state according to financing scheme and number of WoRA (logarithms), 2003 and 2012. (A) Government schemes, 2003. (B) Government schemes, 2012. (C) Social security, 2003. (D) Social security, 2012. Although government schemes improved the alignment of expenditure with potential demand from 2003–2012, a high degree of variability persists among states.

The financial gap among states narrowed for government schemes, with a maximum/minimum ratio of 103.8/4.1 (25.3) in 2003 versus 288.4/24.3 (11.86) in 2012 (S1 Appendix). There were, however, significantly larger disparities between spending levels in different states for the population without social security (Fig 6A and 6B). In the social security-funded population, the relationship between expenditure and potential demand remained stable (Fig 6C and 6D).

The government schemes tightened the alignment between expenditure and potential demand during the period from 2003 to 2012. However, a high degree of variability persists among states.

## Discussion

This past decade, Mexico has witnessed a significant transformation in the level and distribution of MHFP expenditure. These changes flowed directly from the policies implemented between 2003 and 2015, designed to address the fifth MDG and improve access to MHFP services alongside major health system reforms. Policies were focused mainly on women without access to social security, and designed to provide financial protection for the poorest households. Our results show that the changes in expenditure were consistent with the financial objectives of the MHFP policies, although not all of these objectives have been met.

Overall, from 2008 onward, public spending on the population without social security increased continually, with a consequent drop in OOP expenditures. These changes proved even more pronounced than those reported for total health expenditure in Mexico [21], due to the existence of synergistic effects following the introduction of the SSPH and the *Embarazo Saludable* strategy. Our findings concur with those of other studies suggesting that the SSPH has boosted both the enrollment of pregnant women in the *SP* and the use of publicly-funded MHFP services [31,32].

The results of this study also demonstrate that, between 2003 and 2012, social security expenditure on MHFP activities decreased by 10%, despite a 38% growth in the total budget for the social security scheme [21]. This can be explained by the 9% reduction that occurred during the period analyzed in the number of hospital inpatient days related to childbirth (vaginal and cesarean), and to the treatment of complications during pregnancy and childbirth [33]. Future studies will be required to determine the effects of these changes on the quality of the services delivered.

The combination of more spending by government schemes and less by social security clearly accounts for the increased financial parity between the two. However, spending levels vary widely among SESAs. This is partly due to the persistence of traditional budgeting practices accounting for 64% of total health expenditure. This may end up countering the effects of the newly introduced SSPH allocation mechanisms, which have enabled government schemes to improve their alignment of fresh resources with potential demand. Purchasing decisions concerning goods and services also vary a great deal among the public financing schemes. While these decisions are taken at the federal level for social security, they are made at the state level for government schemes. In the case of *SP*, problems in accountability between state and federal government have been reported [34–36]. Future studies should analyze whether changes in financial allocation criteria improve resource administration and efficiency.

The results also show that households without social security experienced greater reductions in OOP expenditure than their counterparts. This is particularly true for childbirth services. It can therefore be assumed that expanded coverage of such services at the institutional level contributes to the financial protection of these households. Various studies have pointed out the central role of *SP* in increasing the use of public hospital services [37,38], especially for childbirth [32], and in enhancing the financial protection for households using services from the MoH [39,40].

The shift in public spending towards prevention rather than treatment of pregnancy and childbirth complications reflects the government’s efforts to improve MHFP service coverage [32,41,42]. The apparently contradictory rise in public spending on complications can be explained by recent decisions to increase the number of *SP* affiliates and the launch of the Comprehensive Strategy for Reducing Maternal Mortality.

While many important improvements have been made in MHFP funding, various studies in Mexico have pointed out that the increase in institutionalized childbirth services does not appear to be linked to an adequate reduction in either maternal mortality or qualified obstetric care [32]. Research has also shown that gaps in the delivery of effective pregnancy and childbirth services persist in some states, particularly for vulnerable populations [42]. The need to improve coverage for these services and enhance availability of contraception is clear [41–43]. Achieving better maternal health outcomes requires not only increases in public spending on services, but also effective targeting to ensure the adoption of preventive actions in family planning and antenatal care. Pregnancy and childbirth services also need improvement.

The RHS are designed to collect the best and most complete financial data on reproductive health. They have their limitations, however, especially their sources of information. Analyzing household expenditure on family planning relies on a biannual household survey centered only on the purchase of family planning methods. We incorporated data from other sources, particularly the National Reproductive Health Survey, to complete this information. Financial data, particularly on government schemes, are not standardized, mainly because of the newly installed SSPH, but also as a result of delays and possible misreporting at the state level. This could cause errors in the assessment and comparability of public funding information. Seeking to reduce possible bias, we interviewed financial officers in each institution, and collected additional data to improve the quality of analysis.

The financial changes resulting from the policies implemented between 2003 and 2012 suggest that Mexico is on the right track. It is imperative, however, to reflect on the internal and external factors influencing resource allocation and expenditure efficiency (i.e. SESA mechanisms for purchasing and decision-making, political changes and economic policies), while also considering the need to modify such factors in the short and medium term to improve state-level performance. More in-depth analysis is required to determine the association between state-level MHFP expenditure and effective coverage of services, and to identify opportunities for improvement in maternal health policies.

## Conclusions

The establishment and continuous development of RHS in Mexico over the past 10 years have made it possible to demonstrate their usefulness in charting the financial panorama of reproductive health at the national level. RHS have also contributed to clarifying the financial consequences of policies. As policies are developed to take the MDGs beyond 2015 [44], the RHS are useful in examining the resource flows related to these goals. The Commission on Information and Accountability for Women’s and Children’s Health [45] has recommended analysis of resource flows as the first step towards measuring the financial impact of MHFP policies. The second step is to analyze the achievements in health outcomes and contrast them with investments [46]. This requires a commitment from governments to strengthening their health accounting systems and creating new instruments to measure outcomes and better understand the consequences of policies on their target populations.

The global forums currently defining the Sustainable Development Goals and Post-2015 Development Agenda [47] have stipulated that actions to improve the health of women and families must play a central role in public policy. Within this context, tracking of resources in healthcare should be a means to assess accountability and a fundamental tool for evidence-based decision-making and resource allocation.

## Supporting Information

S1 Appendix Maternal health and family planning expenditure per woman of reproductive age in Mexico, 2003–2012, by state and financial scheme. 2012 PPP USD.
(DOCX)
