# Supplementary material for: Financing Maternal Health and Family Planning: Are We on the Right Track? Evidence from the Reproductive Health Subaccounts in Mexico, 2003–2012
Source: PLoS One. 2016 Jan 26;11(1):e0147923. doi: 10.1371/journal.pone.0147923 (PMC4728114; doi:10.1371/journal.pone.0147923)
Supplement: S1 Appendix — 2012 PPP USD. (DOCX) [file pone.0147923.s001.docx]

**S1 Appendix. Maternal health and family planning expenditure per woman of reproductive age in Mexico, 2003-2012, by state and financial scheme. 2012 PPP USD**

| **State** | **Government schemes** | | **Social security** | |
| --- | --- | --- | --- | --- |
|  | **2003** | **2012** | **2003** | **2012** |
| Aguascalientes | 41.7 | 123.5 | 115.4 | 76.2 |
| Baja California | 24.4 | 38.4 | 111.0 | 97.4 |
| Baja California Sur | 103.8 | 79.7 | 168.3 | 165.3 |
| Campeche | 87.8 | 151.8 | 146.2 | 114.9 |
| Coahuila | 73.1 | 288.4 | 105.6 | 82.6 |
| Colima | 102.5 | 58.8 | 128.2 | 108.1 |
| Chiapas | 28.0 | 71.0 | 91.0 | 77.6 |
| Chihuahua | 27.9 | 167.8 | 109.3 | 87.0 |
| Distrito Federal | 38.6 | 113.3 | 136.8 | 138.6 |
| Durango | 45.5 | 110.1 | 117.6 | 107.1 |
| Guanajuato | 9.8 | 27.5 | 125.0 | 109.2 |
| Guerrero | 4.1 | 37.9 | 96.4 | 113.5 |
| Hidalgo | 40.1 | 55.3 | 191.9 | 86.9 |
| Jalisco | 21.3 | 55.5 | 121.0 | 98.2 |
| México | 15.7 | 149.2 | 70.0 | 62.8 |
| Michoacán | 18.2 | 60.7 | 109.1 | 134.6 |
| Morelos | 48.4 | 24.8 | 90.9 | 92.5 |
| Nayarit | 101.5 | 139.4 | 164.4 | 116.8 |
| Nuevo León | 42.3 | 73.3 | 107.9 | 78.2 |
| Oaxaca | 46.3 | 103.0 | 89.6 | 87.8 |
| Puebla | 37.0 | 49.9 | 86.5 | 114.4 |
| Querétaro | n.a | 108.1 | 153.0 | 81.9 |
| Quintana Roo | 86.1 | 24.3 | 127.3 | 87.2 |
| San Luis Potosí | 44.7 | 125.0 | 91.1 | 80.3 |
| Sinaloa | 58.3 | 103.8 | 94.3 | 104.0 |
| Sonora | 61.7 | 136.1 | 85.1 | 86.7 |
| Tabasco | 74.6 | 92.9 | 103.8 | 78.0 |
| Tamaulipas | 12.3 | 139.2 | 77.4 | 63.9 |
| Tlaxcala | 27.4 | 35.6 | 146.4 | 92.9 |
| Veracruz | 17.1 | 53.7 | 81.0 | 65.7 |
| Yucatán | 52.2 | 83.9 | 130.7 | 92.3 |
| Zacatecas | 81.7 | 162.2 | 108.1 | 86.9 |
| **Maximum** | **103.8** | **288.4** | **191.9** | **165.3** |
| **Minimum** | **4.1** | **24.3** | **70.0** | **62.8** |
| **Max/Min** | **25.09** | **11.87** | **2.74** | **2.63** |
